# Novel antiemetic effect of naldemedine in patients initiating opioids for cancer pain: a secondary analysis of a randomized clinical trial

**DOI:** 10.1093/oncolo/oyag047

**Published:** 2026-02-18

**Authors:** Takahiro Higashibata, Takaomi Kessoku, Shinya Kajiura, Mami Hirakawa, Shunsuke Oyamada, Keisuke Ariyoshi, Takeshi Yamada, Yoshiyuki Yamamoto, Toshikazu Moriwaki, Hiroka Nagaoka, Yasuyuki Takashima, Kosuke Doki, Bryan J Mathis, Kosuke Tanaka, Akiko Fuyuki, Atsushi Nakajima, Yo Ishihara, Ryuji Hayashi, Takayuki Ando, Yuko Kobayashi, Naoki Izawa, Yoshiki Horie, Tatsuya Morita, Jun Hamano

**Affiliations:** Department of Palliative and Supportive Care, Institute of Medicine, University of Tsukuba, Tsukuba 305-8575, Japan; Department of Gastroenterology and Hepatology, Yokohama City University Graduate School of Medicine, Yokohama 236-0004, Japan; Department of Gastroenterology, International University Health and Welfare Graduate School of Medicine, Narita 286-8686, Japan; Department of Palliative Medicine and Gastroenterology, International University Health and Welfare Narita Hospital, Narita 286-8520, Japan; Medical Oncology and Palliative Medicine Department, Toyama University Hospital, Toyama 930-0194, Japan; Department of Palliative Medicine, St. Marianna University School of Medicine, Kawasaki 216-8511, Japan; Department of Biostatistics, Japanese Organisation for Research and Treatment of Cancer (JORTC) Data Center, Tokyo 116-0013, Japan; Department of Data Management, JORTC Data Center, Tokyo 116-0013, Japan; Tsukuba Clinical Research & Development Organization (T-CReDO), University of Tsukuba, Tsukuba 305-8576, Japan; Department of Gastroenterology, Institute of Medicine, University of Tsukuba, Tsukuba 305-8575, Japan; Department of Gastroenterology and Hepatology, Kurashiki Central Hospital, Kurashiki 710-8602, Japan; Department of Palliative Care, Tokyo Metropolitan Cancer and Infectious Diseases Center Komagome Hospital, Tokyo 113-8677, Japan; Tsukuba Clinical Research & Development Organization (T-CReDO), University of Tsukuba, Tsukuba 305-8576, Japan; Department of Pharmaceutical Sciences, Institute of Medicine, University of Tsukuba, Tsukuba 305-8575, Japan; Department of Cardiovascular Surgery, Institute of Medicine, University of Tsukuba, Tsukuba 305-8575, Japan; Department of Gastroenterology and Hepatology, Yokohama City University Graduate School of Medicine, Yokohama 236-0004, Japan; Department of Gastroenterology, International University Health and Welfare Graduate School of Medicine, Narita 286-8686, Japan; Department of Palliative Medicine and Gastroenterology, International University Health and Welfare Narita Hospital, Narita 286-8520, Japan; Department of Gastroenterology and Hepatology, Yokohama City University Graduate School of Medicine, Yokohama 236-0004, Japan; Department of Palliative Care, Shinyurigaoka General Hospital, Kawasaki 215-0026, Japan; Department of Gastroenterology and Hepatology, Yokohama City University Graduate School of Medicine, Yokohama 236-0004, Japan; Department of Palliative Medicine and Gastroenterology, International University Health and Welfare Narita Hospital, Narita 286-8520, Japan; Medical Oncology and Palliative Medicine Department, Toyama University Hospital, Toyama 930-0194, Japan; Third Department of Internal Medicine, Faculty of Medicine, Academic Assembly, University of Toyama, Toyama 930-0194, Japan; Department of Pharmacy, St. Marianna University Hospital, Kawasaki 216-8511, Japan; Department of Clinical Oncology, St. Marianna University School of Medicine, Kawasaki 216-8511, Japan; Department of Clinical Oncology, St. Marianna University School of Medicine, Kawasaki 216-8511, Japan; Department of Palliative and Supportive Care, Seirei Mikatahara General Hospital, Hamamatsu 433-8558, Japan; Research Association for Community Health, Hamamatsu 434-0046, Japan; Department of Palliative and Supportive Care, Institute of Medicine, University of Tsukuba, Tsukuba 305-8575, Japan

**Keywords:** naldemedine, OINV, OIC, prevention, RCT

## Abstract

**Background:**

Several studies have suggested that naldemedine may reduce opioid-induced constipation (OIC) as well as opioid-induced nausea and vomiting (OINV). This study aimed to investigate prophylactic effects of naldemedine on OINV in patients initiating regular, oral, strong opioids for cancer pain.

**Methods:**

In this preplanned secondary analysis of a multicenter, double-blind, randomized, placebo-controlled trial investigating the preventive effects of naldemedine on OIC, eligible patients were randomized in a 1:1 ratio to receive either naldemedine 0.2 mg or placebo once daily for 14 days. The primary endpoint was the complete response (CR) rate, defined as the proportion of patients with no vomiting episodes and no use of rescue antiemetics within the first three days of opioid initiation. The secondary endpoint was the nausea and vomiting score of the European Organization for Research and Treatment of Cancer Quality of Life Questionnaire Core 15 Palliative Care (EORTC QLQ-C15-PAL).

**Results:**

Of the 103 patients, 48 and 47 patients in each group started protocol treatment, respectively. The CR rate was significantly higher in the naldemedine group than in the placebo group (81.3% vs 38.3%, *P* < .001). Nausea and vomiting scores on the QLQ-C15-PAL at weeks 1 and 2 were significantly better in the naldemedine group (means 7.1 and 6.4) versus placebo (means 44.6 and 35.3; both *P* < .001). Within the total effect of naldemedine on the QLQ-C15-PAL nausea and vomiting scores at week 2, the proportion mediated through OIC reduction was 21.9%.

**Conclusions:**

Naldemedine may have intrinsic antiemetic potency to prevent both OIC and OINV.

**Trial registration:**

https://jrct.niph.go.jp/ (Japan Registry of Clinical Trials) Identifier: jRCTs031200397

Implications for Practice Concomitant use of naldemedine with opioids prevents opioid-induced constipation. In addition, naldemedine has been demonstrated to possess intrinsic antiemetic potency by blocking the actions of opioids. However, there is no currently reported, randomized, placebo-controlled trial evaluating the antiemetic effect of naldemedine. This secondary analysis of a randomized, placebo-controlled trial showed that more patients in the naldemedine group reported no vomiting episodes and no rescue antiemetic use after opioid initiation than in the placebo group. The findings suggest that naldemedine may have intrinsic antiemetic potency and could potentially prevent both opioid-induced constipation, nausea, and vomiting.

## Introduction

Opioids are essential medications for patients with moderate to severe pain related to cancer or active cancer treatment,^1^ but a substantial number are forced to compromise their pain management by reducing the dose of their opioids, switching opioids, or changing the route of administration due to adverse effects.^2^^,^^3^ One of the major adverse effects of opioids is gastrointestinal dysfunction, such as constipation, bloating, incomplete evacuation, increased gastric reflux, nausea, and vomiting.^4^ Among these, nausea and vomiting is particularly distressing symptoms that can affect overall outcome, medication, compliance, enteral absorption, and quality of life.^5^ The incidence of opioid-induced nausea and vomiting (OINV) has been reported to be 8.3%-18.3% and 22.7%-40.0% in a prospective observational study, and 21.3% and 12.7% in a systematic review, respectively.^6^^,^^7^ OINV usually attenuates within one week of opioid initiation due to tolerance, but is not always a transient or short-term adverse effect.^5^ Notably, 20.7% of patients on long-term opioids for cancer pain reported no attenuation of OINV over time.^8^ The pathogenesis of OINV can be complex, including direct stimulation of the chemoreceptor trigger zone (CTZ) and vestibular apparatus, impaired gastric emptying, and initiation of opioid-induced constipation (OIC).^9–11^

The American Society of Clinical Oncology (ASCO) guideline conditionally recommends pretreatment with metoclopramide or prochlorperazine around the clock for the first few days of opioid therapy for patients reporting previous OINV.^1^ In Europe. 36.3% of European physicians provided antiemetics as around the clock medication to prevent OINV.^12^ However, it has not been established which antiemetics are effective in preventing OINV.^9^ One randomized controlled trial (RCT) showed that the concurrent use of prochlorperazine seems to be ineffective in preventing OINV^13^ while another RCT showed that neither ondansetron nor metoclopramide was significantly more effective than placebo in controlling OINV among patients with cancer, including opioid-naïve patients.^14^ The major palliative care guidelines (other than the ASCO guideline) from the Multinational Association of Supportive Care in Cancer, the European Society for Medical Oncology, and the European Association for Palliative Care recommend metoclopramide, prochlorperazine, and haloperidol as first-line antiemetics for the treatment of OINV, but do not recommend any antiemetics for prevention.^1^^,^^3^^,^^4^^,^^11^

Naldemedine is a peripherally acting µ-opioid receptor antagonist (PAMORA) that reverses OIC by blocking opioid actions at peripheral µ-opioid receptors in the gastrointestinal tract without adversely affecting analgesia.^15^ In addition, recent studies have suggested that naldemedine may also suppress OINV by blocking opioid action in several peripheral tissues. Experiments on animals indicated that naldemedine and the δ-opioid receptor-selective antagonist, TAN-452, suppressed morphine-induced emesis in ferrets.^10^^,^^16^ Naldemedine showed more potent antagonist activity to the human δ-opioid receptor than naloxone and naloxegol in an in vitro functional assay.^15^ In a retrospective observational study, the incidence of OINV was significantly lower in patients who received naldemedine within two days of opioid initiation,^9^ and an RCT comparing naldemedine with magnesium oxide in preventing OIC showed that the incidence of nausea as an adverse event was significantly lower in the naldemedine group.^17^ However, there is no currently reported, randomized, placebo-controlled trial evaluating the antiemetic effect of naldemedine using specific outcomes related to nausea and vomiting. Although our previous report clarified the preventive effect of naldemedine on OIC,^18^ it remains unknown whether the reduction of OIC by naldemedine contributes to the prevention of OINV.

The aim of the present analysis is to evaluate whether prophylactic use of naldemedine at opioid initiation can prevent OINV in patients with cancer pain. Additionally, this study examines whether, and to what extent, there is an indirect effect of naldemedine through OIC reduction, which may subsequently reduce nausea and vomiting after opioid initiation.

## Methods

### Study design

This study is a preplanned secondary analysis of a multicenter, double-blind, randomized, placebo-controlled trial evaluating the preventive effect of naldemedine on OIC in patients with cancer starting regularly scheduled strong opioids.^18^ Patients were recruited at the University of Tsukuba Hospital, the Yokohama City University Hospital, the Toyama University Hospital, and the St. Marianna University School of Medicine between July 2, 2021 and May 30, 2023, and were followed up for 14 days. This study focused preplanned secondary endpoints related to nausea and vomiting after opioid initiation; however, this analysis was not specified in the previously published study protocol.^19^ This study was conducted in accordance with the Declaration of Helsinki and Japan’s Clinical Trials Act. The protocol was approved by the University of Tsukuba Clinical Research Review Board on January 26, 2021 (approval reference number TCRB20-001). Prior to the start of patient enrollment, the study was registered in the Japan Registry of Clinical Trials (jRCT) as jRCTs031200397. This study was reported according to the CONSRT 2010 guidelines^20^ and AGReMA-SF (A Guideline for Reporting Mediation Analyses Short-Form).^21^ Written, informed consent to participate in the study was obtained from all participating patients.

### Subjects

All participants from the original trial were included in this study. Patients enrolled were 20 years or older and first-time users of a regularly scheduled, oral, strong opioid (morphine, oxycodone, or hydromorphone) for cancer pain. Patients with gastrointestinal obstruction and patients who had undergone interventions affecting gastrointestinal function were not eligible. Detailed inclusion and exclusion criteria have been published previously.^19^ After enrollment, eligible patients were randomly assigned in a 1:1 ratio to receive either naldemedine (Symproic^®^ 0.2 mg) or placebo. Randomization was computer-generated using a minimization method with “institution,” “Eastern Cooperative Oncology Group performance status (≤1 or ≥2),” “gastrointestinal or non-gastrointestinal cancer,” and “regular laxative use prior to enrollment (yes or no)” as assignment adjustment factors. Allocation grouping information was available only to the data management officer on the electronic data capture system. Research staff, patients, pharmacists, and statistical analysts were masked throughout the study medication distribution and outcome process.

### Intervention

The protocol treatment period was 14 days after the start of naldemedine or placebo. Regular antiemetic use prior to enrollment was not discontinued. Rescue antiemetic use for nausea and vomiting during the protocol treatment period was permitted in both groups, depending on oncologists and palliative care physicians.

### Measurements

The primary endpoint of this secondary analysis was the complete response (CR) rate for nausea and vomiting within the first three days of opioid initiation. The CR rate was defined as no vomiting episodes and no use of rescue antiemetics within three days.^13^^,^^22^ Previous RCTs set the evaluation period for the prevention of OINV at 24 h and five days;^13^^,^^14^ however, the appropriate evaluation period for the prevention of OINV has not been determined. In this study, the evaluation period was set at three days based on prior finding that OINV typically occurs within 48 h of opioid initiation.^13^ While the proportions of patients who had at least one episode of vomiting and those who used antiemetic drugs within three days of opioid initiation were prespecified in the protocol, the CR rate was designated as the primary endpoint for this secondary analysis after trial registration and the primary publication.

The secondary endpoints were scores for item 9 (nausea and vomiting) of the Japanese version of the European Organization for Research and Treatment of Cancer Quality of Life Questionnaire Core 15 Palliative Care (EORTC QLQ-C15-PAL) at weeks 1 and 2. The QLQ-C15-PAL was developed as a shortened version of the EORTC QLQ-C30 to assess quality of life for cancer patients in palliative care.^23^ Nausea and vomiting are rated together as item 9 on a 4-point Likert scale from 1 (not at all) to 4 (very much). The score was converted to a range of 0 to 100 according to the EORTC scoring manual. Higher scores indicate more severe nausea and vomiting. The validity and reliability of both the Japanese versions of the QLQ-C15-PAL have been confirmed.^24^ A study to determine the minimal important differences (MID) for the QLQ-C15-PAL showed that the MID for improvement in the nausea category was −13.4, though no statistically significant MID for deterioration was established.^25^

OIC was assessed at weeks 1 and 2 using the Bowel Function Index (BFI), which consists of three items that evaluate the ease of defecation, residual stool, and symptoms of constipation over the past seven days on a visual analogue scale from 0 (complete freedom from the symptom) to 100 (highest perceived feeling of the symptom).^26^ An average score of 28.8 or higher on these three BFI items was defined as the development of OIC.^27^

### Statistical analysis

The primary endpoint comparison of the CR rates between groups was conducted according to the intention-to-treat principle, using the chi-squared test and the student’s *t*-test. Stratified analysis was also performed to compare the CR rates between groups using the Cochran–Mantel–Haenszel test with the following stratification factors: constipation, plus nausea, and vomiting at baseline, determined by a BFI score of 28.8 or higher, and a QLQ-C15-PAL nausea and vomiting score of two or higher on the 4-point Likert scale. As a sensitivity analysis, univariate and multivariate logistic regression analyses adjusted for age, sex, gastrointestinal cancer, baseline BFI scores, and baseline QLQ-C15-PAL nausea and vomiting scores were performed to examine the robustness of the findings.

**Figure 1:**
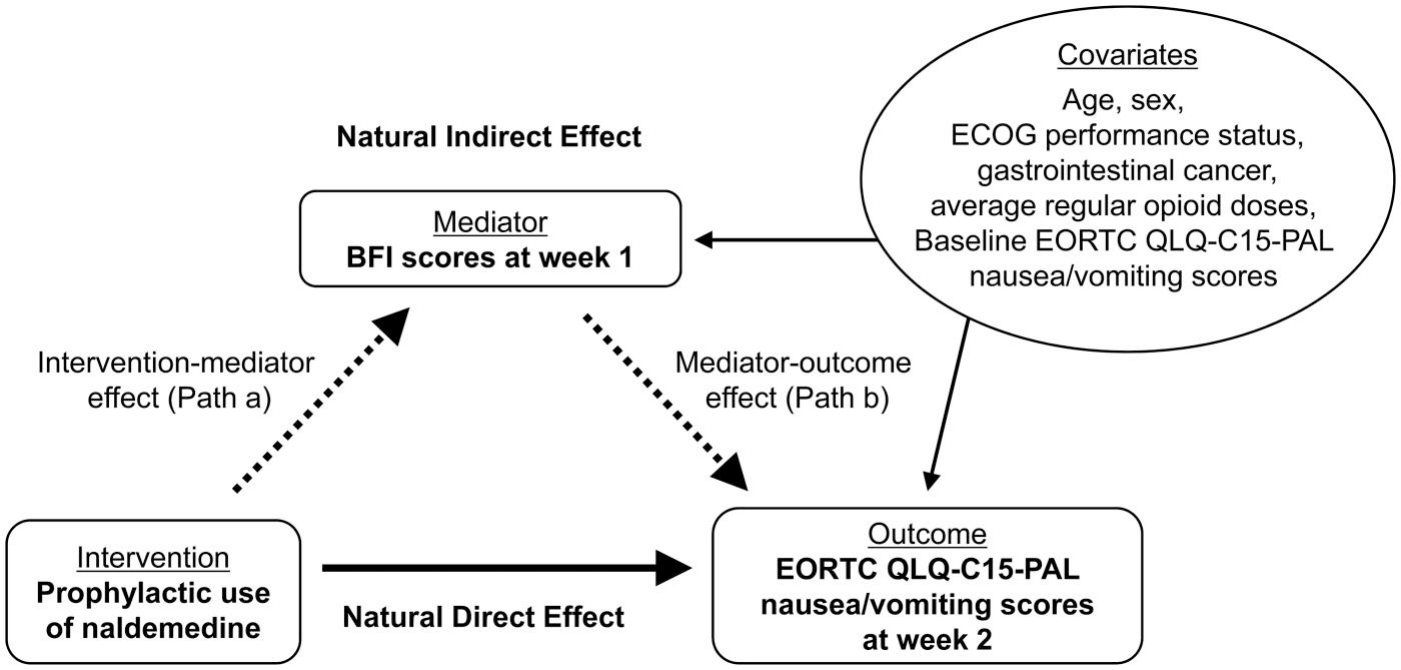
Single mediator model for nausea and vomiting scores on the EORTC QLQ-C15-PAL. The natural indirect effect is represented by the dot lines through the mediator BFI (the combination of path a and b); the natural direct effect is represented by the solid line; The influence of possible confounders is represented by the thin lines. Abbreviations: BFI, Bowel Function Index; ECOG, Eastern Cooperative Oncology Group; EORTC QLQ-C15-PAL, European Organization for Research and Treatment of Cancer Quality of Life Questio1nnaire Core 15 Palliative Care.

Differences in nausea and vomiting scores between groups on the QLQ-C15-PAL were compared using linear regression analysis. Causal mediation analysis was performed to test the single mediator mechanism through OIC reduction on nausea and vomiting scores at week 2 (Figure 1), based on the assumption that naldemedine partly reduces OINV through OIC reduction. Estimates of the natural indirect effect (NIE) as the mediation effect, the natural direct effect (NDE), and the total effect (TE) were calculated using the counterfactual approach. NIE, NDE, and TE were presented as mean difference with 95% confidence interval (CI). The estimand of this analysis was the proportion of the mediation effect of naldemedine, calculated as the ratio of NIE to TE.

BFI scores were treated with a binominal category (BFI scores of 28.8 or higher) as the mediator representing OIC. In the mediator model, the intervention-mediator effect (path a) was analyzed by using logistic regression with BFI scores at week 1 as the dependent variable, treatment assignment as the independent variable, and gender, performance status, gastrointestinal cancer, and baseline BFI scores as covariates based on clinical judgment. In the outcome model, the mediator-outcome effect (path b) was analyzed by using linear regression analysis with QLQ-C15-PAL nausea and vomiting scores at week 2 as the dependent variable, BFI scores at week 1 as the independent variable, and treatment assignment, baseline QLQ-C15-PAL nausea and vomiting scores, age, gender, performance status, gastrointestinal cancer, and average regular opioid dose as covariates based on previous reports.^28^^,^^29^

The sample size was calculated based on the primary endpoint of the original trial (incidence of OIC), and the target number of enrolled patients was set at 100.^18^ A two-tailed *P* value <.05 was considered statistically significant. All analyses were performed using STATA, version 18.0 (Stata Corp., College Station, TX, USA). The “mediate” command was used to compute NIE, NDE, and TE in the causal mediation analysis.

## Results

**Figure 2:**
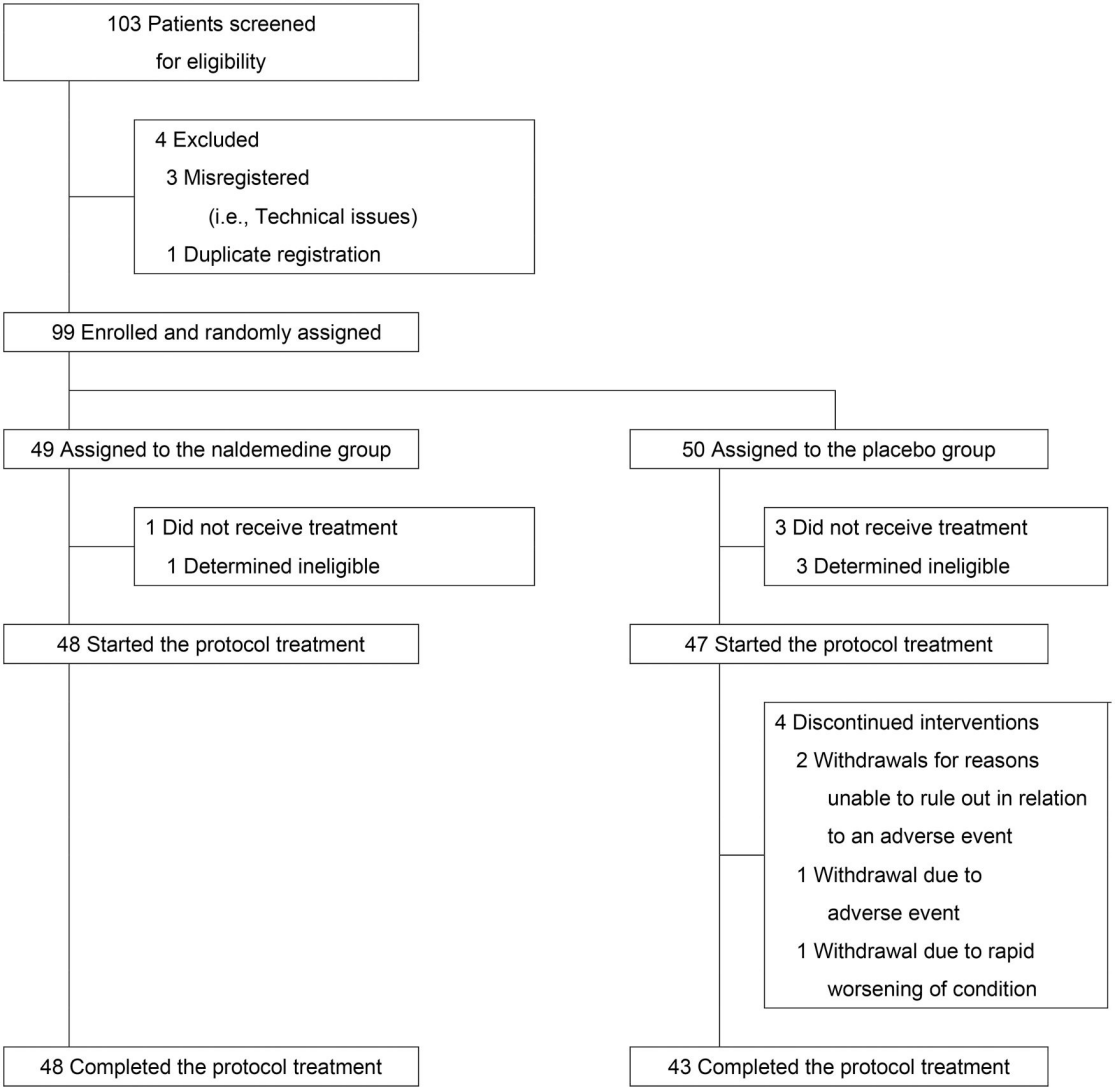
Trial profile.

Of 103 patients screened for eligibility, four patients were excluded due to duplicate registration (*n* = 1) or misregistration (i.e., technical issues, *n* = 3). A total of 99 patients were randomized in a 1:1 ratio to receive either naldemedine (*n* = 49) or placebo (*n* = 50; Figure 2). Finally, 48 and 47 patients in each group started the protocol treatment. Baseline characteristics of the patients are summarized in Table 1. 56.8% of the study patients had hepatobiliary, pancreatic, or gastrointestinal cancer. The characteristics were well balanced between the two groups. Nearly the same proportion of patients used strong opioids as rescue medication and regular weak opioids prior to enrollment. Only one patient in the naldemedine group used regular weak opioids together with strong opioids as rescue medication before enrollment. Regular opioid starting doses at baseline and the average total opioid doses during the study period were higher in the naldemedine group.

**Table 1:** Characterstics of study participants.

	Naldemedine	Placebo
	(*n* = 48)	(*n* = 47)
**Age (years)^a^**	67.9 (11.3)	67.4 (11.0)
**Sex**		
** Female**	25 (52.1)	32 (68.1)
** Male**	23 (47.9)	15 (31.9)
**ECOG performance status**		
** 0**	14 (29.2)	15 (31.9)
** 1**	25 (52.1)	25 (53.2)
** 2**	8 (16.7)	4 (8.5)
** 3**	1 (2.1)	3 (6.4)
**Cancer type**		
** Hepatobiliary and pancreatic**	16 (33.3)	17 (36.2)
** Gastrointestinal**	8 (16.7)	10 (21.3)
** Gynecological**	5 (10.4)	5 (10.6)
** Breast**	4 (8.3)	4 (8.5)
** Genitourinary**	4 (8.3)	4 (8.5)
** Lung**	4 (8.3)	3 (6.4)
** Other**	7 (14.6)	4 (8.5)
**Metastatic site**		
** No metastases**	24 (50.0)	23 (48.9)
** Present**		
** Lung**	11 (22.9)	8 (17.0)
** Liver**	8 (16.7)	8 (17.0)
** Bone**	10 (20.8)	7 (14.9)
** Peritoneal dissemination**	3 (6.3)	9 (19.1)
** Central nervous system**	0 (0.0)	0 (0.0)
** Other**	9 (18.8)	11 (23.4)
**History of gastrointestinal tract surgery**	14 (29.2)	12 (25.5)
**Strong opioids starting at enrollment**		
** Oxycodone**	42 (87.5)	42 (89.4)
** Hydromorphone**	6 (12.5)	5 (10.6)
**Strong opioids used prior to enrollment as rescue use only**	11 (22.9)	11 (23.4)
**Regular weak opioids used prior to enrollment**	7 (14.6)	7 (14.9)
**Regular laxatives used prior to enrollment**	17 (35.4)	14 (29.8)
**Regular opioid starting dose at baseline(mg/day)^a, b^**	18.3 (8.5)	15.7 (2.9)
**Average regular opioid dose during the study period (mg/day)^a, b^**	19.6 (8.7)	16.2 (3.7)
**Average rescue opioid dose during the study period (mg/day)^a, b^**	3.1 (5.4)	3.0 (4.1)
**Average total opioid dose during the study period (mg/day)^a, b^**	22.7 (10.2)	19.1 (6.1)
**EORTC-QLQ-C15-PAL nausea/vomiting at baseline**		
** Not at all**	42 (87.5)	32 (68.1)
** Little**	5 (10.4)	10 (21.3)
** Quite a bit**	1 (2.1)	3 (6.4)
** Very much**	0 (0.0)	1 (2.1)
** Unknown**	0 (0.0)	1 (2.1)

Abbreviations: ECOG, Eastern Cooperative Oncology Group; EORTC QLQ-C15-PAL, European Organization for Research and Treatment of Cancer Quality of Life Questionnaire Core 15 Palliative Care.

^a^Mean (standard deviation).

^b^Oral morphine equivalent daily dose.

In contrast, the proportion of female patients and those with nausea and vomiting at baseline was higher in the placebo group.

The CR rate was significantly higher in the naldemedine group than in the placebo group (81.3% vs 38.3%, % difference [95% CI], 43.0% [25.2 to 60.7]; *P* < .001; Table 2). These significant differences in the CR rates persisted even when patients with constipation and nausea and vomiting at baseline were excluded. Stratified analysis also confirmed the superiority of naldemedine over placebo in the CR rate (*P* < .001; Table 2). A sensitivity analysis supported the robustness of the finding that a greater proportion of patients in the naldemedine group achieved a CR compared with those in the placebo group (Supplementary Table S1—see online supplementary material, odds ratio = 8.79; 95% CI, 3.01-25.70).

**Table 2:** CR rate for nausea and vomiting within three days of opioid initiation.

	Naldemedine	Placebo	% Difference	*P* ^a^	*P* ^b^
**Total patients**	*n* = 48	*n* = 47			
**CR rate, % (95% CI)**	81.3 (70.2 to 92.3)	38.3 (24.4 to 52.2)	43.0 (25.2 to 60.7)	<.001	<.001
** Female**	*n* = 25	*n* = 32			
** CR rate, % (95% CI)**	68.0 (49.7 to 86.3)	28.1 (12.5 to 43.7)	39.9 (15.9 to 63.9)	.003	
** Male**	*n* = 23	*n* = 15			
** CR rate, % (95% CI)**	95.7 (87.3 to 104.0)	60.0 (35.2 to 84.8)	35.7 (9.5 to 61.8)	.006	
** Patients without constipation at baseline**	*n* = 35	*n* = 35			
** CR rate, % (95% CI)**	82.9 (70.4 to 95.3)	34.3 (18.6 to 50.0)	48.6 (28.5 to 68.7)	<.001	
** Patients with constipation at baseline**	*n* = 13	*n* = 12			
** CR rate, % (95% CI)**	76.9 (54.0 to 99.8)	50.00 (21.7 to 78.3)	26.9 (−9.4 to 63.3)	.161	
** Patients without nause/vomiting at baseline**	*n* = 42	*n* = 32			
** CR rate, % (95% CI)**	81.0 (69.1 to 92.8)	34.4 (17.9 to 50.8)	46.6 (26.3 to 66.9)	<.001	
** Patients with nausea/vomiting at baseline**	*n* = 6	*n* = 15			
** CR rate, % (95% CI)**	83.3 (53.5 to 113.2)	46.7 (21.4 to 71.9)	36.7 (−2.4 to 75.7)	.125	

Abbreviations: CI, confidence interval; CR, complete response.

^a^Chi-square test.

^b^Cochran–Mantel–Haenszel test for data stratified according to sex, constipation at baseline, and nausea/vomiting at baseline.

**Figure 3:**
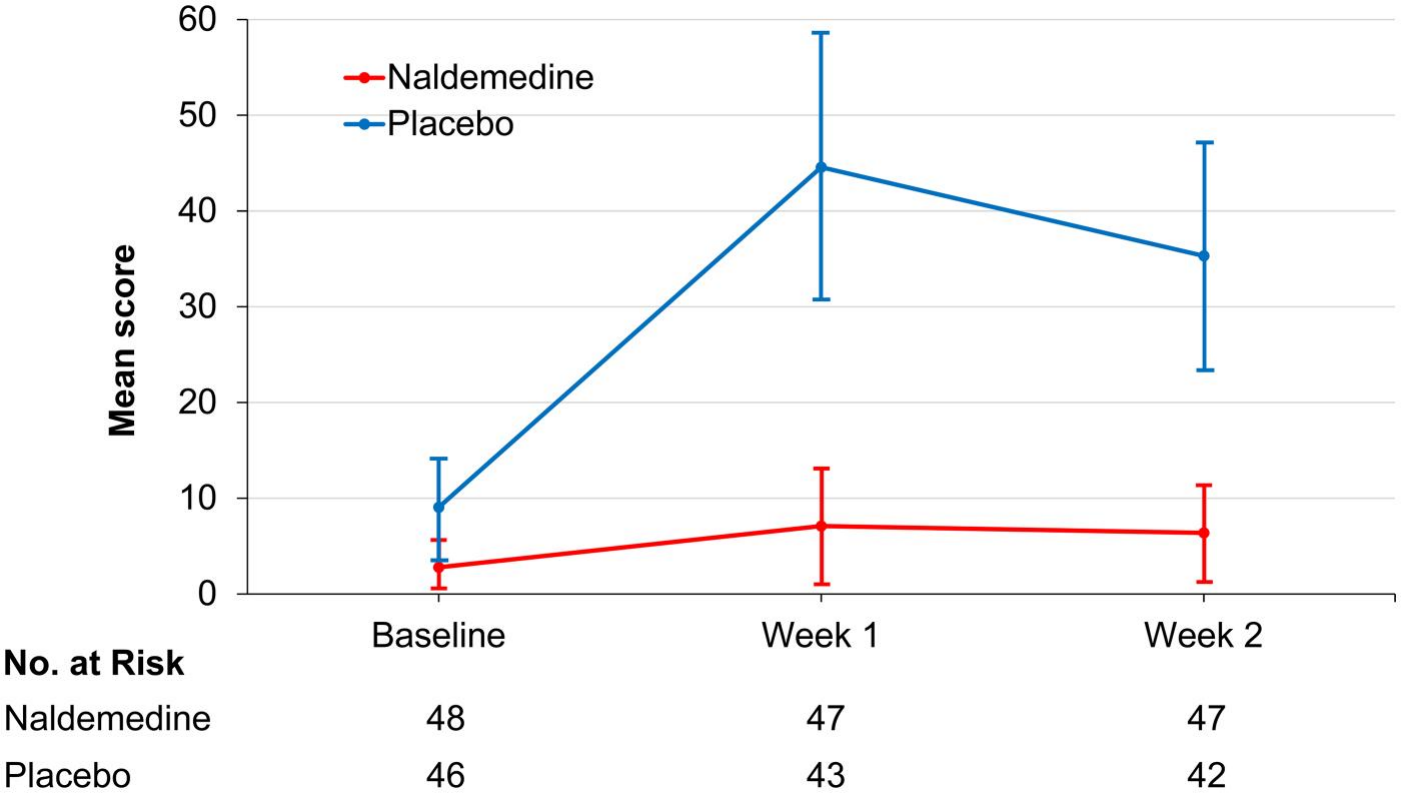
Line plot of mean nausea and vomiting scores of the EORTC QLQ-C15-PAL for naldemedine and placebo groups at baseline, week 1, and week 2. Data are presented as mean (95% confidence interval). EORTC QLQ-C15-PAL indicates European Organization for Research and Treatment of Cancer Quality of Life Questionnaire Core 15 Palliative Care.

The mean (95% CI) scores for nausea and vomiting on the QLQ-C15-PAL at weeks 1 and 2 were 7.1 (1.0 to 13.2) and 6.4 (1.1 to 11.6) for naldemedine and 44.6 (30.9 to 58.3) and 35.3 (23.5 to 47.1) for placebo, respectively, with statistically significant differences (both *P* < .001; Figure 3). Nausea and vomiting scores for naldemedine remained low from baseline to week 2, while the scores for placebo worsened over two weeks, peaking at week 1. When comparing scores between groups, naldemedine significantly mitigated the worsening of scores compared to placebo.

Causal mediation analysis showed that within the TE of naldemedine on nausea and vomiting scores at week 2, NIE through OIC reduction was −5.7 (95%CI, −11.3 to −0.1) and NDE was −20.2 (95% CI, −34.9 to −5.5). The mediation effect through OIC reduction accounted for 21.9% of the TE, while the proportion of the effects attributable to mechanisms other than the reduction of OIC was 78.1% (Table 3).

**Table 3:** Effects of naldemedine on EORTC QLQ-C15-PAL nausea/vomiting score.

	Week 2
**EORTC QLQ-C15-PAL nausea/vomiting score**	
** Naldemedine, *n***	46
** Placebo, *n***	42
** Intervention-mediator effect (path a), OR (95% CI)**	2.0 (0.9 to 3.1)
** Mediator-outcome effect (path b), MD (95% CI)**	−22.4 (−39.8 to −5.0)
** NIE, MD (95% CI)**	−5.7 (−11.3 to −0.1)
** NDE, MD (95% CI)**	−20.2 (−34.9 to −5.5)
** Total effect, MD (95% CI)**	−25.9 (−38.8 to −13.0)
** Proportion mediated**	21.9%

Abbreviations: CI, confidence interval; EORTC QLQ-C15-PAL, European Organization for Research and Treatment of Cancer Quality of Life Questionnaire Core 15 Palliative Care; MD, mean difference; NDE, natural direct effect; NIE, natural indirect effect; OR, odds ratio.

## Discussion

This study showed that prophylactic use of naldemedine in patients starting regularly scheduled, oral, strong opioids for cancer pain significantly reduced the incidence of nausea and vomiting within the first three days of opioid initiation. This is the first double-blind, randomized, placebo-controlled trial to demonstrate an effective antiemetic in preventing OINV. Naldemedine may have intrinsic antiemetic potency and could potentially prevent both OIC and OINV.

The most important finding is that patients starting with naldemedine had significantly higher CR rates regarding nausea and vomiting within three days of opioid initiation than those starting with placebo. This finding is in line with the OINV-related outcomes reported in our previous study, which showed lower proportions of patients experiencing at least one episode of vomiting and of those using antiemetic medication within three days of opioid initiation in the naldemedine group.^18^ Compared to previous research, which reported a five-day CR rate of 69.5% for prochlorperazine with no significant difference from placebo (69.5% vs 63.3%),^13^ the three-day CR rate of 81.3% for naldemedine in this study is 40% higher than placebo, with a significant difference. This difference between the drugs may be attributable to the limitation of prochlorperazine in blocking multiple mechanisms of OINV. Conversely, opioids cause nausea and vomiting mainly by stimulating opioid receptors outside the blood–brain barrier, which naldemedine could antagonize.

This interventional study included patients with nausea and vomiting at enrollment. Nausea and vomiting scores on the QLQ-C15-PAL at baseline were significantly higher in the placebo group than in the naldemedine group. In addition, nausea and vomiting after the start of the intervention were not always due to opioids because of causative factors other than opioids, such as recent chemotherapy, radiotherapy, brain metastases, or electrolyte imbalances. However, these factors were unlikely to have had a significant impact on the outcome of the study as the difference in the CR rates between groups remained large and statistically significant (46.6%) even after excluding patients who still had nausea and vomiting at baseline. Stratified analysis and multivariate logistic regression analysis further suggested that baseline nausea and vomiting did not appear to substantially confound the preventive effect of naldemedine on OINV. Similarly, the use of strong and weak opioids prior to enrollment is unlikely to have confounded the results, as these factors were well balanced between the two groups. Although regular opioid starting doses at baseline were higher in the naldemedine group than in the placebo group, this would likely bias the results toward a lower CR rate in the naldemedine group.

The second important finding is that the proportion of the mediation effect of naldemedine through OIC reduction on nausea and vomiting after opioid initiation was 21.9%. This also indicated that approximately 80% of the antiemetic effect of naldemedine within the first two weeks of opioid initiation can be attributed to its direct effect. The multiple and complex mechanisms underlying OINV have previously hindered the identification of effective antiemetics for OINV; however, naldemedine may have intrinsic antiemetic potency for OINV by blocking the multiple actions of opioids, including stimulation of the CTZ, sensitization of the vestibular apparatus, impaired esophageal and gastric motility, and delayed gastric emptying.^5^ Nevertheless, approximately 20% of patients on naldemedine failed to achieve a CR after opioid initiation. To further reduce the incidence of OINV, further studies are needed to determine the mechanisms by which naldemedine reduces OINV and the characteristics of patients with OINV despite prophylactic use of naldemedine.

This study has several limitations. First, this was a secondary analysis of the randomized, placebo-controlled trial designed primarily to assess the preventive effect of naldemedine on OIC. The CR rate was not prespecified in the protocol and was one of several secondary endpoints. Therefore, this study was exploratory, and further RCTs focusing on OINV as the primary outcome are needed for validation. Second, the study population consisted of Japanese patients in a specific setting. Thus, these facts require cautious interpretation when generalizing the findings to other ethnic populations and settings. Third, the observed effects may partly reflect baseline imbalances between the groups, particularly in nausea and vomiting, even though randomization was performed. Fourth, this study did not record the regular use of medications with antiemetic effects, such as metoclopramide, domperidone, histamine H1 receptor antagonists, antipsychotics, and corticosteroids, prior to enrollment. Regular antiemetic medication during the study period may have increased the CR rates in both groups. An imbalance between the groups in the proportion of patients receiving regular antiemetic medication, as well as laxatives or opioids, could have introduced co-intervention bias. Fifth, in the causal mediation analysis, the QLQ-C15-PAL nausea and vomiting scores at week 2 were set as the outcome, although the scores at week 1 may have been more relevant to evaluate the preventive effect of naldemedine on OINV. The scores at week 2 were chosen to follow the mediator (BFI scores at week 1). Sixth, the causal mediation analysis is generally based on a strong set of identification assumptions, including no unmeasured confounding and no mediator-outcome confounding affected by the exposure. Although a series of confounders were adjusted for in this study, the possibility of residual confounding cannot be ruled out. Seventh, this study evaluated the preventive effects of naldemedine on OINV but did not investigate the therapeutic effects. Therefore, further studies are warranted to clarify whether naldemedine can improve OINV once it occurs. Finally, this study had a relatively short evaluation period of two weeks. Thus, it is necessary to investigate the long-term efficacy for OINV when naldemedine is administered prophylactically.

In conclusion, this study suggests the novel antiemetic effect of naldemedine for OINV prophylaxis. Further RCTs are warranted to validate these results, given the high risk of bias in this study. The preventive effects of naldemedine on both OIC and OINV would be a unique feature not found in other laxatives for OIC. By preventing both OIC and OINV in patients initiating opioids for cancer pain, prophylactic use of naldemedine may enhance patient compliance and improve overall pain management.

## Supplementary Material

oyag047_Supplementary_Data

## Data Availability

The authors declare that all data supporting the findings of this study are available within the article and its appendix. Researchers can apply for data by submitting a proposal to the corresponding author.
